# Understanding the Mechanism of Bent DNA Amplifying Sensors Using All-Atom Molecular Dynamics Simulations

**DOI:** 10.3390/bios15050272

**Published:** 2025-04-26

**Authors:** Kaitlin Bullard, Deborah Okyere, Shelbi J. Foster, Asmaa A. Sadoon, Jiali Li, Jingyi Chen, Yong Wang

**Affiliations:** 1 Department of Physics, University of Arkansas, Fayetteville, AR 72701, USA; 2 Materials Science and Engineering Program, University of Arkansas, Fayetteville, AR 72701, USA; 3 Department of Chemistry and Biochemistry, University of Arkansas, Fayetteville, AR 72701, USA; 4 Department of Physics, University of Thi-Qar, Nassiriya 64001, Iraq; 5 Cell and Molecular Biology Program, University of Arkansas, Fayetteville, AR 72701, USA

**Keywords:** DNA bows, melting curve, FRET, NAMD, VMD

## Abstract

Bent DNA amplifying sensors were recently developed to amplify and quantify the interactions of DNA with various salts and molecules. However, a thorough quantitative understanding of their mechanism is missing. Here, using all-atom molecular dynamics (MD) simulations, we investigate the behavior and dynamics of sharply bent DNA molecules in the absence and presence of Mg^2+^ ions at different concentrations. The simulations show that Mg^2+^ ions reduce the fluctuations of DNA strands, enhance base-pairing, and stabilize bent DNA molecules. The computational results are further verified by both melting curve experiments and ensemble FRET measurements, highlighting the mechanical instability and sensitivity of bent DNA molecules.

## 1. Introduction

DNA is one of the most fundamental components of life, and its interactions with various molecules are critical for regulating cellular processes, understanding disease mechanisms, and advancing drug development [1,2,3,4,5,6,7,8,9,10,11,12,13,14,15]. Additionally, many processes in cells require bending DNA sharply [16,17,18,19,20,21,22,23,24,25]. As a result, various techniques have been developed to understand the interactions of DNA with other molecules [26,27,28,29,30,31,32,33,34,35,36,37,38,39,40,41,42,43,44], and extensive efforts have been made to investigate the flexibility and mechanics of bending DNA [45,46,47,48,49,50,51,52].

On a different note, bent DNA structures have also been employed in other applications. For example, bent double-stranded DNA molecules were used as molecular springs to exert force on proteins, providing a means to mechanically control the functions of proteins [53,54,55,56,57]. Bent DNA molecules were also applied to aptamers to measure their folding energy [58]. Recently, we demonstrated that sharply bent DNA structures can significantly amplify the interactions of DNA with various salts and organic molecules [59,60]. This approach offers a sensitive and cost-effective method for detecting and quantifying DNA interactions with other molecules, showing potential for both fundamental research and biomedical applications [59,60]. The proposed mechanism of bent DNA amplifying sensors is that pre-bending the double-stranded DNA molecules makes them more unstable; thus, they are more sensitive to perturbations [59,60].

In this study, we aim to quantitatively understand the behavior and dynamics of sharply bent DNA amplifying sensors and test the proposed hypothesis on their working mechanism. We adopt the classical all-atom molecular dynamics (MD) approach, in which all the atoms of the DNA and the surrounding solvent and ions are computed based on effective interactions [61]. This approach has been extensively used for studying short segments of DNA [61,62,63,64,65]. Recently, Cong et al. employed it to revisit the anomalous bending elasticity of sharply bent DNA and reported the importance of pre-existing nicks in DNA bending mechanics [66].

While more than a dozen salts and organic molecules have been tested experimentally for bent DNA amplifying sensors [60], we select Mg^2+^ ions as an example in this study to examine their effects on the behavior and dynamics of sharply bent DNA molecules using all-atom MD simulations, because Mg^2+^ ions are well known for DNA replication, repair, and stabilization [26,28,67,68]. MD simulations show that sharply bent DNA molecules display higher fluctuations, larger base-pair distances, and fewer hydrogen bonds in the absence of Mg^2+^ ions. In contrast, the presence of Mg^2+^ ions reduces strand fluctuations and enhances base-pairing, stabilizing bent DNA molecules. The computational results from the MD simulations are then verified experimentally using both DNA melting curve experiments and ensemble fluorescence resonance energy transfer (FRET) measurements, confirming our hypothesis of the working mechanism of bent DNA amplifying sensors.

## 2. Materials and Methods

### 2.1. DNA Sequence and Initial Conformation for MD Simulations

**Figure 1:**
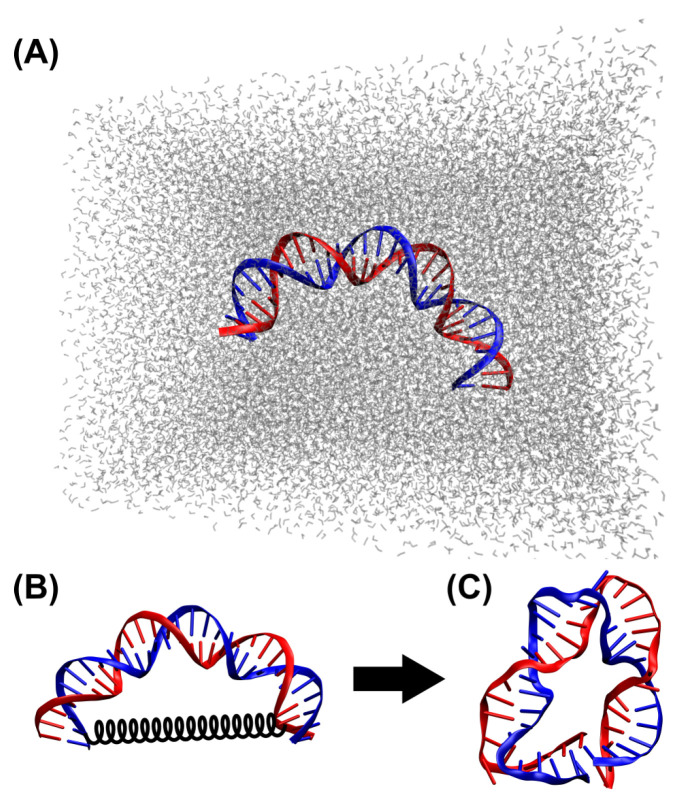
MD simulation set-up in this study. (**A**) Initial conformation of double-stranded DNA of 30 bp solvated in a water box. (**B**) A virtual spring (black) was introduced to connect the 2nd and 29th residues/bases to further bend the DNA in the MD simulations. (**C**) An example of the DNA conformation at the end of the MD simulation for 260 ns. The DNA strands and bases are shown in blue and red in all panels.

The 30 bp sequence of the bent DNA bows from our previous work [59,60] was used in the MD simulations in this study. Briefly, strand B has a sequence of 5′ - CTG CTG AAT TCT GTG GAG TCG TCG TAT GTC - 3′, while the sequence of the complementary strand (strand C) is 3′ - GAC GAC TTA AGA CAC CTC AGC AGC ATA CAG - 5′. The initial conformation of the double-stranded DNA was generated by X3DNA [69,70,71], with the global bending angle set to 160° (Figure 1A). The produced DNA sequence from X3DNA was further cleaned in VMD (version 1.9.3), followed by generating the PSF and PDB files for MD simulations using psfgen [72].

### 2.2. MD Simulations

The DNA was first solvated in a water box of ∼90 Å × 120 Å × 90 Å, followed by adding Na^+^ and Cl^−^ ions at 10 mM (neutralized), as shown in Figure 1A. Then, Mg^2+^ ions were added (in the form of MgCl_2_ salt) at various concentrations: 0 (control), 1, 10, 100, and 1000 mM. The concentrations of the Mg^2+^ ions were much higher than those in the experiments [59,60] and physiological concentrations of 15–25 mM [73], because the time scales of MD simulations were much shorter than the experiments. High concentrations of Mg^2+^ ions, in the order of M or sub-M, have been previously used in MD studies [74,75]. Both solvation and ionization were carried out using VMD plugins [72]. The simulations were performed using NAMD (version 2.13, multicore, GPU accelerated) [76] with CHARMM force fields [77]. To further bend the DNA molecule, a virtual spring was introduced to connect C6 carbon atoms of the 2nd (residue/base T) and 29th (residue/base T) bases of strand B using the tclForces feature of NAMD [76], as shown in Figure 1B. The spring constant of the virtual spring was k=0.04 kcal/mol/Å^2^, while the equilibrium length of the spring was set to 0 Å. Additionally, to avoid the whole molecule moving out of the water box during the MD simulations, the C6 carbon atom of the 2nd residue/base (T) was fixed. Each MD simulation was carried out for 260 ns (after minimization with 10,000 steps) using periodic boundary conditions at a constant temperature of 300 K [66]. Following Cong et al. [66], an NVT ensemble was chosen in all the simulations.

### 2.3. Trajectory Analysis

The trajectories of the bent DNA molecules were analyzed using VMD (version 1.9.3) [72] and MDAnalysis (a Python package; version 2.7.0) [78,79]. The root mean square deviations (RMSD) were calculated using all the atoms of the DNA molecule by MDAnalysis [78,79]. For the root mean square fluctuation (RMSF) analysis, the DNA trajectory of each simulation was first aligned to the last frame to obtain the average structure and then re-aligned to the average structure. The RMSF values for each atom of the DNA molecule were calculated by MDAnalysis [78,79], while the RMSF trajectories of the C6 and P atoms of the DNA molecule were visualized. The base-pair distances of the sharply bent DNA molecules were evaluated using (a) the distances between the C6 atoms of the base-pairs, $\Delta_{C6}$, and (b) the Watson–Crick base-pair distances (i.e., the distances of the N1 and N3 atoms), $\Delta_{WC}$. Additionally, we counted the number of hydrogen bonds in the sharply bent DNA molecules using the HBonds plugin of VMD, with a cutoff distance between the donor and acceptor of 3.0 Å, a cutoff angle of 20°, and only considering polar atoms (N, O, S, and F) [72].

### 2.4. Melting Curve Experiments

For the melting curve experiments, sharply bent DNA amplifying sensors from our previous work were used [59,60]. One strand is strand B, while the other strand (strand CS, 45 bases) has a sequence of 5′ - CAC AGA ATT CAG CAG CAG GCA ATG ACA GTA GAC ATA CGA CGA CTC - 3′ [59,60]. The left 1/3 of the strand CS hybridizes to the left half of strand B, while the right 1/3 of strand CS hybridizes to the right half of strand B, leaving the middle 1/3 of strand CS unhybridized [46,59,60]. After hybridization, strand B (and its complementary segment of strand CS) would be bent. For comparison, a linear, partially double-stranded DNA molecule was used by hybridizing the middle 30 bases of strand CS with a 30-base strand (strand CL). For each set (bent DNA sensors or linear counterpart), the two strands were mixed at equal molar amounts in the background buffer (0.4 mM Tris-HCl with pH adjusted to 7.5 and 0.5 mM NaCl) to reach a final concentration of 2 μM [59,60], supplemented with Mg^2+^ ions at various concentrations (0, 50, 100, 200, and 400 μM). The mixture was heated to and incubated at 75 °C for 5 min, followed by gradual cooling down to 22 °C (room temperature) over 5 h [59,60]. The mixture was then further incubated at room temperature overnight [59,60]. On the next day, the melting curves of the sharply bent DNA sensors and the linear DNA counterpart were measured by reading the absorption at 260 nm on a Beckman Coulter DU 800 spectrophotometer. For each melting curve, the temperature increased from 20 °C to 80 °C. The ramping rate of the temperature was 0.5 °C/min. The reading average time was 1 s, while the reading interval was 0.5 °C. During the melting curve measurements, the samples were kept in quartz cuvettes, which were sealed by polytetrafluoroethylene (PTFE) stoppers to prevent evaporation.

### 2.5. FRET Measurements

**Figure 2:**
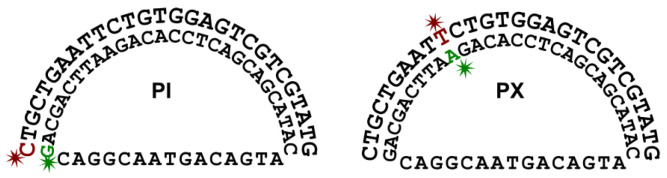
Two sets of locations for fluorescence labeling (green for Cy3, and red for Cy5). PI: Cy5 is attached to residue/base #1 of strand B. PX: Cy5 is attached to residue/base #10 of strand B.

For the FRET experiments, the same DNA strands for the melting curve experiments were used (i.e., strand B and strand CS) [59,60]. However, the two strands were fluorescently labeled by the Cy3/Cy5 pair (purchased from Integrated DNA Technologies) at two different sets of locations (at residue/base #1 or #10 of strand B, as shown in Figure 2). For each set of labeling locations, the two strands were mixed at equal molar amounts in the background buffer (0.4 mM Tris-HCl with pH adjusted to 7.5 and 0.5 mM NaCl) to reach a final concentration of 2 μM [59,60], supplemented with Mg^2+^ ions at various concentrations (0, 1, 2, 4, 8, 16, 31, 63, 125, 250, 500, and 1000 μM). The mixture was heated to and incubated at 75 °C for 5 min, followed by gradual cooling down to 22 °C (room temperature) over 5 h. The mixture was then further incubated at room temperature overnight [59,60]. On the next day, the samples were diluted in the background with corresponding concentrations of Mg^2+^ ions to reach a final concentration of 50 nM, followed by immediately measuring the fluorescence spectra of the diluted samples using a fluorospectrometer (excitation = 500 nm).

## 3. Results

### 3.1. MD Simulations

**Figure 3:**
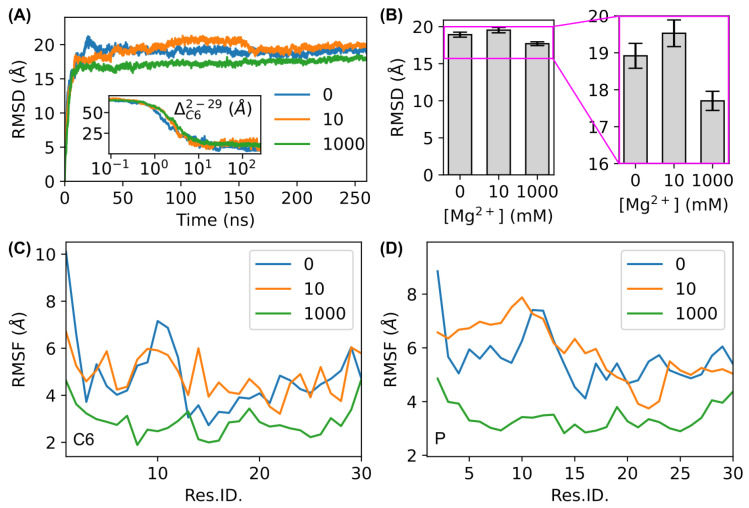
RMSD and RMSF of the MD simulations. (**A**) RMSD of the simulations with Mg^2+^ ions at different concentrations, from 0 mM (control) to 1000 mM. Inset: distance between the C6 atoms of residue 2 and 29 (where the virtual spring is attached), $\Delta_{C6}^{2-29}$, as a function of time. (**B**) Average RMSD of the last 100 ns of the simulations at different concentrations of Mg^2+^ ions. The region highlighted by the magenta box is zoomed in to show the difference. (**C**,**D**) RMSF of the residues/bases (Res.ID) for the (**C**) C6 atoms and (**D**) P atoms, respectively, at different Mg^2+^ concentrations (in mM) from the full trajectories of simulations.

We first analyzed the structural dynamics of the bent DNA amplifying sensors using root mean square deviation (RMSD) and root mean square fluctuation (RMSF). As shown in Figure 3A, the RMSD curves of the DNA molecule with Mg^2+^ ions increased quickly in ∼10 ns and then became stable, indicating that the simulations converged. The first ∼10 ns corresponded to the shrinkage process of the virtual spring attached to residues 2 and 29, as the distance between the C6 atoms of these two residues $\Delta_{C6}^{2-29}$ decreased to stable values in ∼10 ns (inset of Figure 3). We observed that the RMSD values of the DNA sensors remained consistently lower with 1000 mM Mg^2+^ ions than those with 0 or 10 mM Mg^2+^ ions (Figure 3A). Comparing the average RMSD values from the last 100 ns of the simulations showed that this difference was outside of the error bar (Figure 3B). Additionally, we observed slightly larger transitions in the RMSD trajectories at lower concentrations of Mg^2+^ ions (Figure 3A). Similar to the RMSD values, the RMSF values of C6 atoms and P atoms were consistently lower in the presence of Mg^2+^ ions at 1000 mM than the control (Figure 3C,D). The decrease in RMSF values in the presence of Mg^2+^ ions was consistent with a previous study on straight double-stranded DNA [75] and the well-known stabilization effects of Mg^2+^ ions on DNA molecules [26,28,67,68].

**Figure 4:**
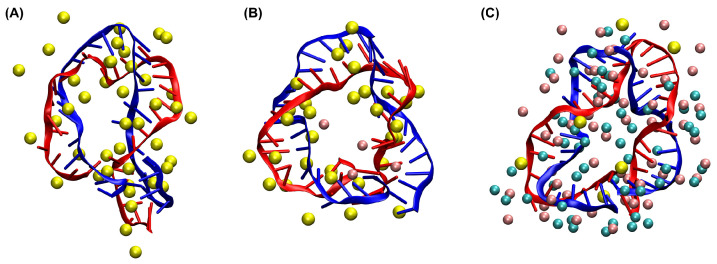
Conformation of the sharply bent DNA amplifying sensors at the end of the simulations of 260 ns with Mg^2+^ ions at different concentrations: (**A**) 0 mM, (**B**) 10 mM, and (**C**) 1000 mM. The DNA strands and bases are shown as blue and red, while the ions within 10Å of the DNA are shown as colored spheres (Na^+^: yellow; Mg^2+^: pink; Cl^−^: teal).

Representative conformations of the sharply bent DNA amplifying sensors at the end of the MD simulations of 260 ns are shown in Figure 4. We observed various “defects” (e.g., disruptions of the base-pairing and helical structure) of the DNA molecule when it was sharply bent in the absence of Mg^2+^ ions (Figure 4A). In contrast, the base-pairing and helical structure were much better preserved in the presence of Mg^2+^ ions at 1000 mM. This visual examination of the final conformation of the bent DNA sensors again confirms that Mg^2+^ ions stabilize DNA molecules.

**Figure 5:**
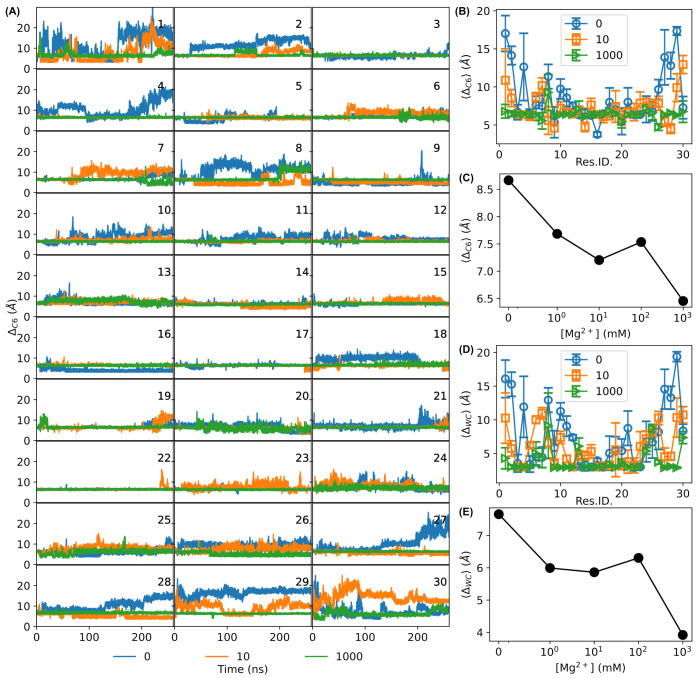
Base-pair distances characterized by (**A**–**C**) the distances between C6 atoms $\Delta_{C6}$ or (**D**,**E**) the Watson–Crick base-pair distance $\Delta_{WC}$. (**A**) Trajectories of distance between C6 atoms of each base-pair $\Delta_{C6}$ of the bent DNA molecule in the absence (blue curves) or presence of Mg^2+^ ions at 10 mM (orange curves) and 1000 mM (green curves). (**B**) Average distance between C6 atoms of each base-pair $\langle \Delta_{C6}\rangle$ calculated from the last 100 ns of the simulations in the absence (blue circles) or presence of Mg^2+^ ions at 10 mM (orange squares) and 1000 mM (green triangles). (**C**) Dependence of the average distance between C6 atoms $\langle \Delta_{C6}\rangle$ of the bent DNA molecule (calculated from the last 100 ns of the simulations) on the concentration of Mg^2+^ ions. (**D**) Average Watson–Crick base-pair distances of each base-pair $\langle \Delta_{WC}\rangle$ calculated from the last 100 ns of simulations in the absence (blue circles) or presence of Mg^2+^ ions at 10 mM (orange squares) and 1000 mM (green triangles). (**E**) Dependence of the average Watson–Crick base-pair distance $\langle \Delta_{WC}\rangle$ of the bent DNA molecule (calculated from the last 100 ns of the simulations) on the concentration of Mg^2+^ ions.

To further quantify the visual observations from the conformations in Figure 4, we calculated the base-pair distances using (a) the distance between C6 atoms of the nucleotides of each base-pair (Figure 5A), $\Delta_{C6}$, and (b) the distance of the nitrogen atoms in the Watson–Crick hydrogen bond, $\Delta_{WC}$. Large variations at both ends of the DNA molecule were present in the absence of Mg^2+^ ions (Figure 5A). Interestingly, in addition to the ends, residues around #10 showed relatively high values and large variations (Figure 5A). In contrast, the base-pair distance trajectories of the molecule in the presence of 1000 mM Mg^2+^ ions showed lower values and smaller variations (Figure 5A). We also quantified the average base-pair distances from the last 100 ns of the simulations (Figure 5B,D). In the absence of Mg^2+^ ions, sharply bent DNA showed large average values for both $\Delta_{C6}$ and $\Delta_{WC}$ at the ends of the bent DNA sensors (Figure 5B,D). For example, $\langle \Delta_{C6}\rangle$ reached ≳15Å of the sharply bent DNA for residues #1 and #2, much greater than that of normal B-DNA (∼6.4 Å), as shown in Figure 5B. With 10 mM Mg^2+^ ions, the base-pair distances lowered, although they remained high at the ends (Figure 5B). In contrast, the base-pair distances became much flatter for the sharply bent DNA sensors in the presence of 1000 mM Mg^2+^ ions, with the majority of residues showing $\langle \Delta_{C6}\rangle \approx 6.5$ Å (Figure 5B), similar to that of normal B-DNA. The Watson–Crick distances showed similar results (Figure 5D). Additionally, averaging the base-pair distances for all the residues/bases of the bent DNA sensors showed that they decreased as the concentration of Mg^2+^ ions increased (Figure 5C,E). These observations confirm that Mg^2+^ ions stabilize the base-pairing of sharply bent DNA amplifying sensors.

**Figure 6:**
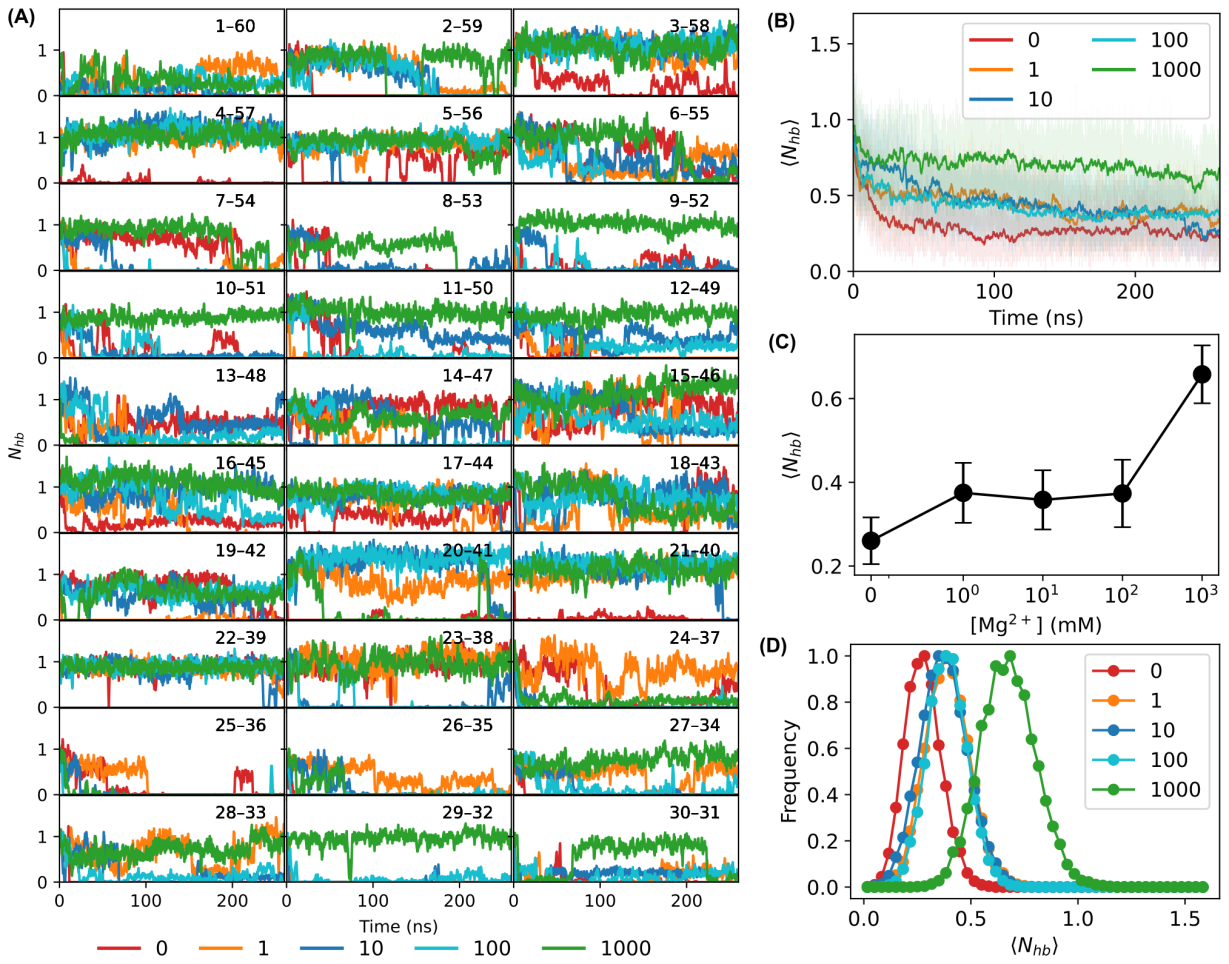
Number of hydrogen bonds per base-pair. (**A**) Time trajectories (smoothed over a window of 101 frames) of the number of hydrogen bonds for each base-pair with Mg^2+^ ions at different concentrations (in mM). (**B**) Average number of hydrogen bonds per base-pair of the bent DNA molecule as a function of simulation time. Solid lines represent a smoothed version of the raw data (averaged over a window of 101 frames). (**C**) Dependence of the average number of hydrogen bonds per base-pair of the bent DNA molecule (calculated from the last 100 ns of the simulations) on the concentration of Mg^2+^ ions. (**D**) Distributions of the average numbers of hydrogen bonds per base-pair of the bent DNA sensors with Mg^2+^ ions at different concentrations (in mM), calculated from the last 100 ns of the simulations. The concentrations of Mg^2+^ ions are indicated by different colors in panels A, B, and D: red, 0 mM; orange, 1 mM; blue, 10 mM; cyan, 100 mM; green, 1000 mM.

The stabilizing effects of Mg^2+^ ions can also be seen from the number of hydrogen bonds formed in the sharply bent DNA sensors. The hydrogen bonds were identified by VMD with a cutoff distance of 3 Å and a cutoff angle of 20°, and the number of hydrogen bonds for each base-pair was counted. As shown in Figure 6A, although the number of hydrogen bonds, $N_{hb}$, showed large variations during the simulations and for different base-pairs, $N_{hb}$ was generally lower without Mg^2+^ ions (red curves) than those with Mg^2+^ ions (e.g., green and cyan curves). The most obvious difference could be seen at residues/bases #3, #4, #8–#12, #16, #17, #20, #21, and #27–#30 (Figure 6A). Taking base-pairs 4–57 and 21–40 as examples, almost no hydrogen bonds were formed in the bent DNA molecule without Mg^2+^ ions, while the number of hydrogen bonds was stable with Mg^2+^ ions. On the other hand, we note that some base-pairs showed low numbers of hydrogen bonds and large fluctuations even with 1000 mM Mg^2+^ ions (e.g., #13 and #24–#26). Therefore, to obtain a better view of the effects of Mg^2+^ ions on the number of hydrogen bonds in the bent DNA molecules, we calculated the average number of hydrogen bonds per base-pair $\langle N_{hb}\rangle$ and examined the distributions of the average number of hydrogen bonds per base-pair under different conditions. As shown in Figure 6B, the average number of hydrogen bonds decreased in the first ∼30 ns of the simulations. After 100 ns, the average number of hydrogen bonds in the bent DNA molecule became stable for all the simulations. However, the average number of hydrogen bonds per base-pair was much higher in the presence of 1000 mM Mg^2+^ ions than that of the control (0 mM Mg^2+^ ions). Calculating the average number of hydrogen bonds per base-pair for all the residues/bases of the DNA molecule from the last 100 ns of the simulations showed that it increased as the concentration of Mg^2+^ ions increased (Figure 6C). Additionally, we plotted the distributions of $\langle N_{hb}\rangle$ at different concentrations of Mg^2+^ ions from the last 100 ns of the simulations (Figure 6C). The distributions appeared as bell-shaped peaks, and the peak shifted to higher values as the concentration of Mg^2+^ ions increased (Figure 6D).

**Figure 7:**
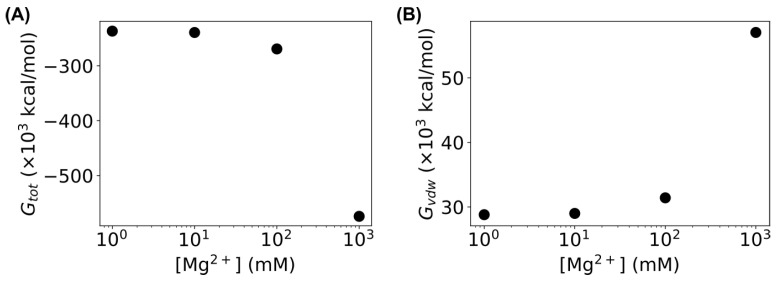
Dependence of the (**A**) total energy and (**B**) van der Waals energy of the bent DNA molecules on the concentration of Mg^2+^ ions. Error bars are smaller than the symbols.

Moreover, we examined the energies in MD simulations of the sharply bent DNA amplifying sensors at different concentrations of Mg^2+^ ions. The energies were extracted from the log files of the MD simulations, and the last 200 ns of the simulations were averaged. As shown in Figure 7A, the total energy ($G_{tot}$) decreased as the concentration of Mg^2+^ ions increased, indicating that the bent DNA molecules became more stable as the concentration of Mg^2+^ ions increased. Additionally, the van der Waals energy ($G_{vdw}$) of the bent DNA molecule increased at higher concentrations of Mg^2+^ ions (Figure 7B), consistent with the increasing number of hydrogen bonds, as observed in Figure 6.

**Figure 8:**
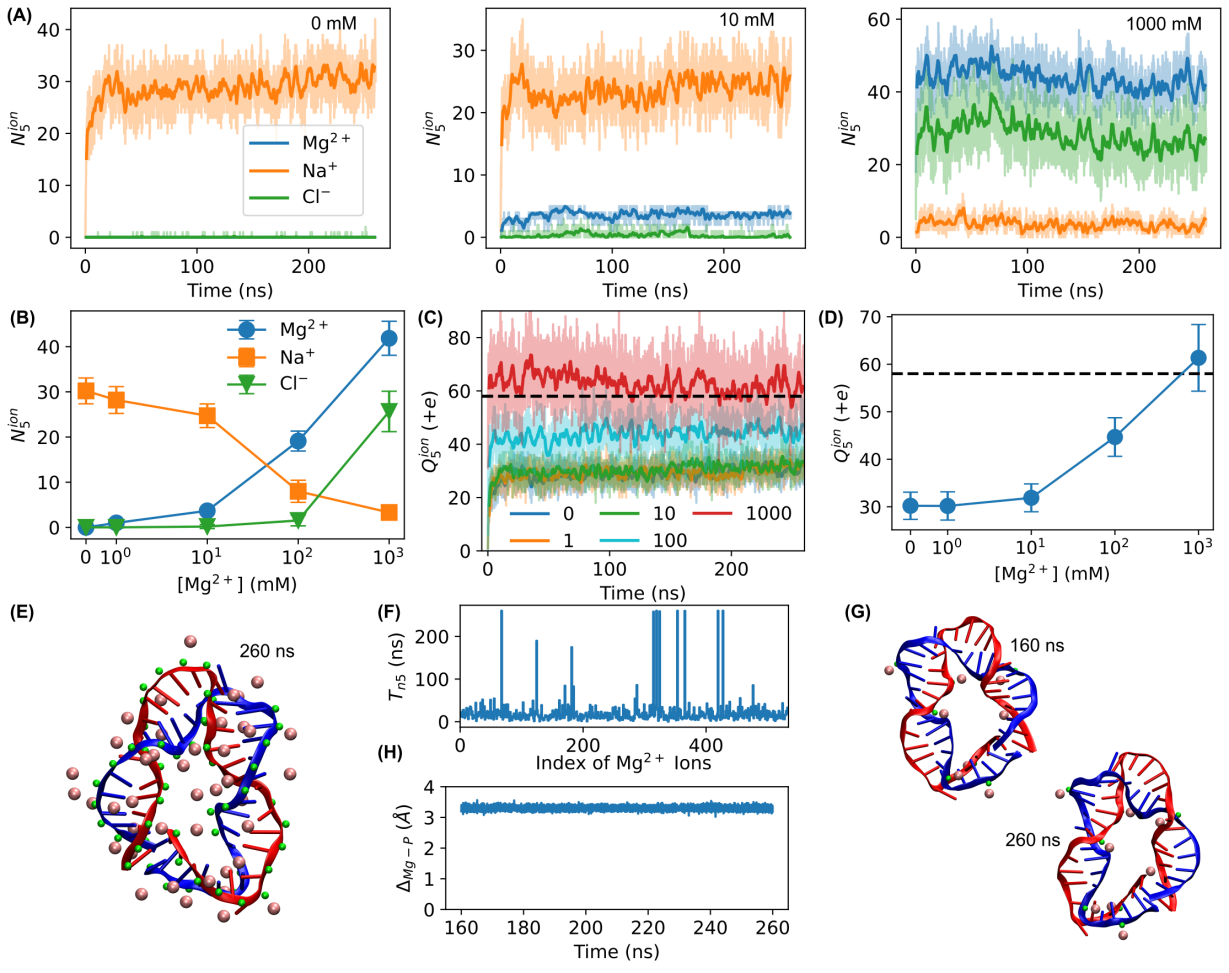
Ions at a proximity of 5 Å of the bent DNA molecule. (**A**) The numbers of ions $N_{5}^{ion}$ (Mg^2+^: blue curves, Na^+^: orange curves, and Cl^−^: green curves) at a proximity of 5 Å of the DNA as functions of simulation time, in the presence of Mg^2+^ ions at different concentrations (0, 10, and 1000 mM). (**B**) Dependence of the average numbers of ions $N_{5}^{ion}$, calculated from the last 100 ns of the simulations, on the concentration of Mg^2+^ ions. (**C**) The total charges from the ions $Q_{5}^{ion}$ at a proximity of 5 Å of the DNA as functions of simulation time in the presence of Mg^2+^ ions at different concentrations (0–1000 mM). The dashed black line indicates the negative charge of the DNA molecule. (**D**) Dependence of the average total charge from the ions, calculated from the last 100 ns of the simulations, on the concentration of Mg^2+^ ions. The dashed black line indicates the negative charge of the DNA molecule. (**E**) Snapshot of Mg^2+^ ions (pink spheres) at a proximity of 5 Å of the bent DNA at the end of the simulation (260 ns), with the phosphorus atoms of the DNA highlighted as green spheres. (**F**) Total duration of each Mg^2+^ ion remaining at a proximity of 5 Å of the DNA, $T_{n5}$, in the presence of 1000 mM Mg^2+^ ions. (**G**) Examples of Mg–P pairs (Mg: pink spheres, and P: green spheres) remaining stable during the last 100 ns of the simulation (with 1000 mM Mg^2+^ ions). (**H**) The distance of an Mg–P pair (atom indices = 1082 and 83330 for P and Mg, respectively) remaining constant in the last 100 ns of the simulation with 1000 mM Mg^2+^ ions.

As charge neutralization by Mg^2+^ ions plays an essential role in stabilizing the base-pairing and stacking of DNA [80,81,82], we examined the number of ions at a proximity of 5 Å of the bent DNA amplifying sensors. We observed that, without or at low concentrations of Mg^2+^ ions, the Na^+^ ions increased quickly and remained at a proximity of the DNA as counterions (Figure 8A). However, with 1000 mM Mg^2+^ ions, the Na^+^ ions were replaced by the Mg^2+^ ions (Figure 8A). Quantifying the average number of ions from the last 100 ns of the simulations showed that the number of Mg^2+^ ions at a proximity of 5 Å of the DNA molecules increased as the bulk concentration of Mg^2+^ ions increased, while Na^+^ ions showed the opposite (Figure 8B). Although the number of Cl^−^ ions in proximity to 5 Å of the DNA also increased at high Mg^2+^ concentrations (Figure 8A,B), we found that the total charge at the proximity of 5 Å of the DNA, $Q_{5}^{ion}=2N_{5}^{Mg}+N_{5}^{Na}-N_{5}^{Cl}$, increased at higher concentrations of bulk Mg^2+^ ions (Figure 8C,D). With 1000 mM Mg^2+^ ions, the total charge from the ions at a proximity of the DNA balanced the negative charge of the DNA molecule (Figure 8C,D). Visualizing all the nearby Mg^2+^ ions and phosphorus atoms of the DNA indicated that some Mg^2+^ ions appeared close to the phosphorus atoms (Figure 8E), supporting that Mg^2+^ ions function as the counterions of negative phosphate groups of DNA. A closer look at each Mg^2+^ ion showed that some Mg^2+^ ions stayed at a proximity of the DNA molecule for long durations (∼100 ns or even >200 ns, as shown in Figure 8F). Examining those Mg^2+^ ions remaining in a proximity of the DNA showed that some of them formed lasting Mg–P pairs (Figure 8G). Quantifying the distance of a Mg–P pair from the last 100 ns of the simulation showed that it remained constant at 3.30±0.05 Å (Figure 8H). These results confirmed that charge neutralization by Mg^2+^ ions plays an important role in the stabilization of bent DNA amplifying sensors.

### 3.2. Melting Curve Experiments

The all-atom MD simulations of the bent DNA sensors supported our hypothesis that pre-bending double-stranded DNA molecules makes them less stable, showing higher fluctuations, larger base-pair distances, and fewer hydrogen bonds in the absence of Mg^2+^ ions. To experimentally verify these computational results, we measured the melting curve of the bent DNA sensors and compared it with that of its linear counterpart. Note that its linear counterpart shared the same CS strand (45 bases) as the bent DNA sensors, while the complementary strand (strand CL) had the same length (30 bases) as strand B of the bent DNA sensors. This strategy resulted in the same length of double-stranded segments for the bent DNA and linear DNA if the strands hybridized perfectly, allowing us to compare the melting curves to identify the difference in hybridization.

**Figure 9:**
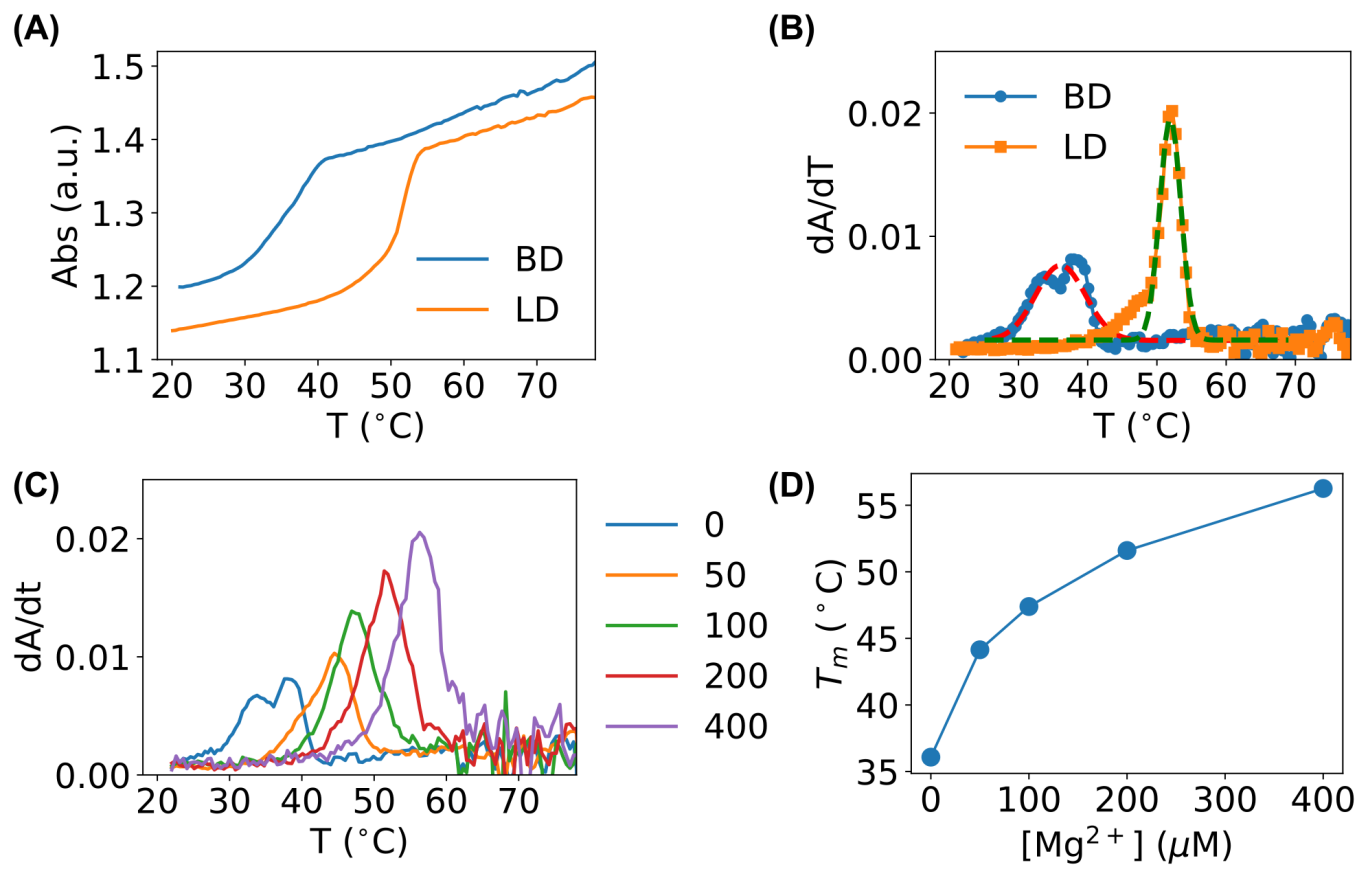
Melting curve comparison between sharply bent DNA sensors (BD) and linear DNA (LD). (**A**) Absorption (wavelength = 260 nm) of the sharply bent DNA sensors (BD) and the corresponding linear DNA (LD) at the same concentration as the temperature increases. (**B**) Derivative of the absorption curves (dA/dt) to determine the melting temperatures ($T_{m}$). Dashed lines are fit with a Gaussian function. (**C**) Melting curves (dA/dt) of the bent DNA sensors in the presence of Mg^2+^ ions at different concentrations (in μM). (**D**) Dependence of the melting temperature of the bent DNA sensors on the concentration of Mg^2+^ ions.

As shown in Figure 9A, both the bent DNA sensors and linear counterpart showed classical S-shaped curves. However, the melting curve of the bent DNA sensors shifted to the left by ∼ 15 °C compared to the linear DNA (Figure 9A), indicating that the bent DNA sensors had a lower melting temperature than their linear counterpart. To see this difference more clearly, we differentiated the absorption–temperature curves (Figure 9B), and the melting temperatures ($T_{m}$) could be obtained from peak centers. Fitting the experimental data provided $T_{m}\approx 36$ °C for the bent DNA sensors and $T_{m}\approx 52$ °C for their linear counterparts. The lower melting temperature confirmed that the bent DNA sensors became more unstable compared to the linear DNA.

The MD simulations showed that Mg^2+^ ions stabilize bent DNA sensors. To experimentally examine this prediction, we measured the melting curves of the bent DNA sensors in the presence Mg^2+^ ions at different concentrations (from 0 to 400 μM). It was confirmed that, as the concentration of Mg^2+^ ions increased, the peaks shifted to higher temperatures (Figure 9C), and the melting temperatures of the bent DNA sensors increased (Figure 9D). At 400 μM Mg^2+^ ions, the melting temperature of the bent DNA sensors became higher (≈56 °C) than that of their linear counterpart.

### 3.3. FRET Measurements

**Figure 10:**
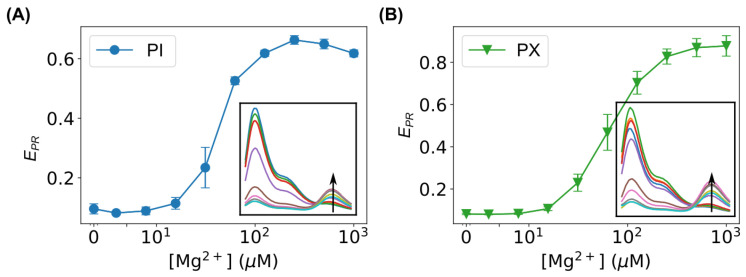
Experimentally measured FRET proximity ratios, $E_{PR}$, of the bent DNA with Mg^2+^ ions at different concentrations (0, 1, 2, 4, 8, 16, 31, 63, 125, 250, 500, and 1000 μM) when labeled at (**A**) residue #1 and (**B**) residue #10. Insets are the corresponding fluorescence spectra used for calculating the FRET proximity ratios, while the arrows indicate the trend of the increasing concentration of Mg^2+^ ions.

To further verify the predictions from the MD simulations, we performed ensemble FRET measurements on fluorescently labeled bent DNA amplifying sensors using a fluorospectrometer. Two labeling constructs were used. The first construct was labeled at the end of the bent DNA strand (i.e., Cy5 at residue #1 of strand B and Cy3 at its complementary base on strand CS). The fluorescence spectra are shown in the inset of Figure 10A for the bent DNA with Mg^2+^ ions at different concentrations (from 0 to 1000 μM). The concentration range of Mg^2+^ ions was chosen to ensure the majority of bent DNA sensors remained as individual bows (i.e., monomers) [59,60] so that we could assess the relative changes in the base-pair distances in the bent DNA structures. As the calculation of the true FRET efficiency requires various corrections (e.g., quantum yields, detector sensitivity, and spectral leakage), it is a common practice to use the proximity ratio to approximate the FRET efficiency [83], $E_{PR}=\frac{F_{A}}{F_{A}+F_{D}}$, where $F_{A}$ is the fluorescence intensity of the acceptor (Cy5) at a wavelength of 670 nm, and $F_{D}$ is the fluorescence intensity of the donor (Cy3) at a wavelength of 570 nm. Due to its simplicity, the FRET proximity ratio was estimated in this study to assess relative changes. As shown in Figure 10A, the FRET proximity ratio ($E_{PR}$) increased as the concentration of Mg^2+^ ions increased, confirming that Mg^2+^ reduced the distance between the donor and acceptor and, thus, the base-pair distance of the bent DNA molecule. For the second construct, we labeled residue #10 of strand B and its complementary base on strand CS. We observed a similar dependence of the FRET proximity ratio on the Mg^2+^ concentration (Figure 10B).

### 3.4. Improvement in Sensitivity of Bent DNA Amplifying Sensors Using FRET-Based Measurements Compared to Gel Electrophoresis

**Figure 11:**
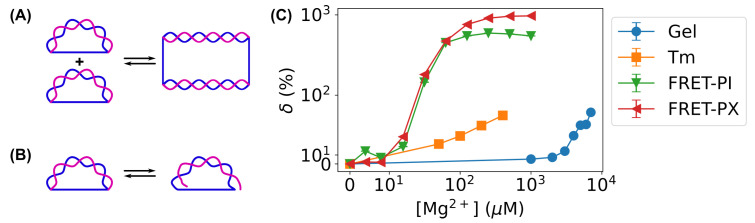
Comparison of sensitivity with different quantification methods. (**A**) Conversion between bent DNA sensors (“monomers”) and straightened, higher-order structures (e.g., “dimers”) detected by gel electrophoresis used in our previous work [59,60]. (**B**) Subtle changes in the hybridization of bent DNA amplifying sensors detected by melting curve and FRET-based methods. Strands of the DNA molecules are shown in blue and magenta in panels A and B. (**C**) Dependence of relative changes in measured quantities in all three types of measurements: blue circles, gel electrophoresis; orange squares, melting curve measurements; green or red triangles, FRET measurements.

In previous studies [59,60], gel electrophoresis was used together with bent DNA amplifying sensors to detect the presence of various ions and organic molecules based on transformations/conversions between bent DNA sensors (“monomers”) to straightened, higher-order structures (e.g., “dimers”, “trimers”, etc.), as shown in Figure 11A. Gel electrophoresis is able to distinguish “monomers” from higher-order structures (“dimers” and “trimers”) but is not capable of detecting the partial dehybridization of bent DNA sensors. Therefore, such transformations in Figure 11A were required for gel electrophoresis when using bent DNA sensors.

In contrast, both the melting curve-based method and the FRET-based method measure the disruption of DNA base-pairing (Figure 5 and Figure 6). Therefore, these methods detect subtler changes in the hybridization of bent DNA amplifying sensors (Figure 11B) compared to gel electrophoresis and, thus, further improve the sensitivity of bent DNA amplifying sensors. This improvement can be seen from the concentration range of Mg^2+^ ions in the current work (≤1 mM) and that in our previous work (≥1 mM) [59,60]. In both studies, the DNA sequences were kept the same. For a more direct comparison, we quantified the relative changes in the measured quantities Q(c) with Mg^2+^ ions at different concentrations (*c*) compared to the control without Mg^2+^ ions [Q(0)], $\delta =∥\frac{Q(c)}{Q(0)}-1∥\times 100\%$. The measured quantities *Q* are the FRET proximity ratios ($E_{PR}$), melting temperature ($T_{m}$), and band intensities of bent DNA bands (*I*) in three types of measurements, respectively. As shown in Figure 11C, the relative changes increased as the concentration of Mg^2+^ ions increased for all three measurement methods. However, the relative changes rose at much lower concentrations of Mg^2+^ ions for the FRET and melting-based methods (Figure 11C). FRET-based measurements were ∼2 orders of magnitude (∼100 times) more sensitive than gel electrophoresis (Figure 11C).

## 4. Conclusions and Discussion

In summary, we exploited all-atom MD simulations to investigate the behavior and dynamics of sharply bent DNA amplifying sensors in the presence of Mg^2+^ ions at different concentrations (from 0 to 1000 mM) in order to understand the mechanism of recently developed bent DNA amplifying sensors. We observed that sharply bent DNA sensors showed higher fluctuations, larger base-pair distances, and fewer hydrogen bonds in the absence of Mg^2+^ ions. In contrast, Mg^2+^ ions significantly reduced fluctuations and enhanced base-pairing, stabilizing the bent DNA molecules. The predictions from the MD simulations were then verified experimentally using both melting curve experiments and ensemble FRET measurements. Results from this study confirmed our previous hypothesis on the mechanism of bent DNA amplifying sensors that sharply bent DNA sensors are closer to breaking down and, thus, more sensitive to the presence of Mg^2+^ ions and other ions and molecules.

Compared to the gel electrophoresis used in our previous work [59,60], the melting curve and FRET-based methods in this work improved sensitivity by 1–2 orders of magnitude. This is because gel electrophoresis-based detection requires the conversion of bent DNA molecules (“monomers”) into higher-order structures (“dimers”, “trimers”, etc.), while melting curve and FRET measurements directly report the relative changes in base-pairing and average base-pair distances.

In the current study, the FRET proximity ratio $E_{PR}$ was used to assess the relative changes in the donor–acceptor distance due to the presence of Mg^2+^ ions at different concentrations, which qualitatively reports the changes in base-pairing of the selected residues of the bent DNA molecule. More sophisticated methods can be used to calculate the true FRET efficiency, and thus the actual donor–acceptor distances, by taking into account and measuring the spectral leakage, detector efficiencies, and quantum yields [84]. On the other hand, if bent DNA molecules are used as biosensors and only relative changes are required, the FRET proximity ratio is a preferred quantity to the true FRET efficiency due to its simplicity and convenience.

In the MD simulations of this work, we placed the Mg^2+^ ions randomly in the water box surrounding the bent DNA molecule, allowed the ions to move “freely”, and explored how they interacted with the bent DNA sensors. This strategy resembled what happened in the experiments; however, high concentrations of Mg^2+^ ions were needed to observe their effects in the short time scales (260 ns) of the all-atom MD simulations. Although this strategy is commonly used in all-atom MD simulations [74,75], an alternative strategy is to restrict Mg^2+^ ions to the proximity of the DNA molecules using forces, which artificially increases the local concentration of the ions. Nonetheless, we expect that the concentrations of Mg^2+^ ions required for visible effects in the simulations will be much higher than those in the experiments due to the mismatch of the time scales.

In this work, Mg^2+^ ions were used as an example in the MD simulations to examine their effects on the sharply bent DNA molecules. Mg^2+^ ions were chosen because it is well known to stabilize double-stranded DNA, thus providing a clear hypothesis to test for sharply bent DNA sensors in MD simulations. We expect that this work is readily applicable to other ions and molecules to understand the mechanisms of bent DNA amplifying sensors for detecting and amplifying their interactions with DNA.

## Data Availability

The data presented in this study are available on request from the corresponding author.
